# Presence of Peripheral Artery Disease Is Associated With Increased Risk of Heart Failure Events: Insights From EMPEROR-Pooled

**DOI:** 10.1161/ATVBAHA.123.319156

**Published:** 2023-05-18

**Authors:** Subodh Verma, Nitish K. Dhingra, Marc P. Bonaca, Javed Butler, Stefan D. Anker, João Pedro Ferreira, Gerasimos Filippatos, James L. Januzzi, Carolyn S.P. Lam, Naveed Sattar, Tomoko Iwata, Matias Nordaby, Martina Brueckmann, Stuart J. Pocock, Milton Packer

**Affiliations:** Division of Cardiac Surgery, St. Michael’s Hospital, University of Toronto, Canada (S.V., N.K.D.).; Division of Cardiology, CPC Clinical Research, University of Colorado School of Medicine, Aurora (M.P.B.).; Baylor Scott and White Research Institute, Dallas, TX (J.B.).; University of Mississippi, Jackson (J.B.).; Department of Cardiology (CVK) of German Heart Center Charité, Institute of Health Center for Regenerative Therapies (BCRT), German Centre for Cardiovascular Research (DZHK), partner site Berlin, Charité Universitätsmedizin, Germany (S.D.A.).; Université de Lorraine, Inserm, Centre d’Investigations Cliniques, Plurithématique 14-33, and Inserm U1116, CHRU, F-CRIN INI-CRCT (Cardiovascular and Renal Clinical Trialists), Nancy, France (J.P.F.).; Department of Surgery and Physiology, Faculty of Medicine of the University of Porto, UnIC@RISE, Cardiovascular Research and Development Center, Portugal (J.P.F.).; Internal Medicine Department, Heart Failure Clinic, Centro Hospitalar de Vila Nova de Gaia/Espinho, Portugal (J.P.F.).; National and Kapodistrian University of Athens School of Medicine, Athens University Hospital Attikon, Greece (G.F.).; Division of Cardiology, Harvard Medical School and Massachusetts General Hospital, Boston (J.L.J.).; National Heart Centre Singapore, Duke-NUS Medical School (C.S.P.L.).; School of Cardiovascular and Metabolic Health, University of Glasgow, Scotland, United Kingdom (N.S.).; Boehringer Ingelheim Pharma GmbH and Co. KG, Biberach, Germany (T.I.).; Boehringer Ingelheim International GmbH, Germany (M.N., M.B.).; First Department of Medicine, Faculty of Medicine Mannheim, University of Heidelberg, Germany (M.B.).; Department of Medical Statistics, London School of Hygiene and Tropical Medicine, United Kingdom (S.J.P.).; Baylor Heart and Vascular Institute, Baylor University Medical Center, Dallas, TX (M.P.).; Imperial College, London, United Kingdom (M.P.).

**Keywords:** heart failure, peripheral arterial disease, sodium-glucose transporter 2 inhibitorscreatinine

While it is well established that peripheral artery disease (PAD) is associated with worsening major adverse cardiovascular events and major adverse limb events,^1^ the relationship between PAD in the context of heart failure (HF) is less well defined.^2^ To this aim, we performed a post hoc analysis of the EMPEROR-Pooled data set to evaluate outcomes of patients with HF across the spectrum of left ventricular ejection fraction by the presence or absence of PAD. We also studied the efficacy and safety of empagliflozin in people with coexistent HF and PAD.

In EMPEROR-Pooled (n=9718), a total of 821 (8.4%) patients had PAD (261 in EMPEROR-Reduced [Empagliflozin Outcome Trial in Patients With Chronic Heart Failure and a Reduced Ejection Fraction] and 560 in EMPEROR-Preserved [Empagliflozin Outcome Trial in Patients With Chronic Heart Failure With Preserved Ejection Fraction]) and 8897 patients did not (3469 in EMPEROR-Reduced and 5428 in EMPEROR-Preserved). Patients with PAD were more likely to be men (70.5% versus 62.6%), White (83.9% versus 72.9%), and older (72.2±8.3 versus 69.7±10.5 years). Patients with PAD were more symptomatic (median Kansas City Cardiomyopathy Questionnaire clinical summary scores [PAD, 68.8 versus no PAD, 75.0]; New York Heart Association class ≥III [PAD, 27.4% versus no PAD, 20.3%]) and were more likely to have ischemic HF (60.5% versus 39.9%), be previous/current smokers (65.0% versus 48.8%), as well as have diabetes (65.2% versus 47.9%), hypertension (90.6% versus 82.9%), and hypercholesterolemia (84.8% versus 62.9%). Left ventricular ejection fraction was similar in patients with and without PAD (45.6±14.5% versus 43.9±15.3%). Patients with PAD had higher systolic blood pressure (131.1±17.1 versus 127.8±16.3 mm Hg), lower eGFR (estimated glomerular filtration rate; 55.6±19.4 versus 61.7±20.6 mL/min per 1.73 m^2^), and a higher proportion of albuminuria (urine albumin-to-creatinine ratio ≥30 mg/g, 51.0% versus 41.6%). With respect to background therapies, patients in both groups had similar rates of angiotensin-converting enzyme inhibitors/angiotensin receptor blockers/angiotensin receptor/neprilysin inhibitor (82.1% versus 83.7%) and β-blocker (90.5% versus 89.4%) use; however, mineralocorticoid receptor antagonist use was lower in the PAD group (43.1% versus 51.2%). Use of other antihypertensives including calcium channel blockers and renin inhibitors (30.1% versus 20.9%), lipid-lowering medications (86.0% versus 69.1%), and antiplatelet therapies (64.7% versus 48.3%) were higher among patients with PAD.

In the pooled analyses, patients randomized to placebo with a history of PAD had an elevated risk of HF outcomes and mortality compared with people without PAD. Specifically, the hazard ratios for total hospitalizations for HF (HHF), cardiovascular death, all-cause mortality, and the composite of cardiovascular death and time to first HHF were higher in people with PAD (total HHF: HR, 1.51 [95% CI, 1.12–2.03]; *P*=0.007; time to cardiovascular death: HR, 1.40 [95% CI, 1.05–1.87]; *P*=0.02; time to all-cause mortality: HR, 1.42 [95% CI, 1.14–1.78]; *P*=0.002; time to first HHF or cardiovascular death: HR, 1.21 [95% CI, 0.98–1.49]; *P*=0.08). Renal outcomes, including slope of eGFR and the composite renal end point, were similar in both PAD and no-PAD patients randomized to placebo in the EMPEROR-Pooled, EMPEROR-Reduced, and EMPEROR-Preserved populations (not shown).

The efficacy of empagliflozin on cardiorenal outcomes in EMPEROR-Pooled was consistent regardless of PAD history (Figure) (PAD: HR for total HHF, 0.64 [95% CI, 0.42–0.98] versus no PAD: HR for total HHF, 0.73 [95% CI, 0.63–0.84]; *P*_interaction_, 0.56). Since patients with PAD were at higher absolute risk, the associated absolute risk reductions for total HHF events was 6.0% amongst patients with PAD and 3.2% amongst those without PAD. The efficacy of empagliflozin was consistent across all other cardiovascular outcomes and concordant in both the EMPEROR-Reduced and EMPEROR-Preserved populations individually. In terms of patient-reported outcomes, empagliflozin increased the Kansas City Cardiomyopathy Questionnaire clinical summary scores by a similar magnitude irrespective of PAD status (adjusted mean difference in EMPEROR-Pooled at week 52: PAD, 2.82 [95% CI, 0.38–5.26]; no PAD, 1.46 [95% CI, 0.73–2.20]; *P*_interaction_, 0.30).

**Figure.**
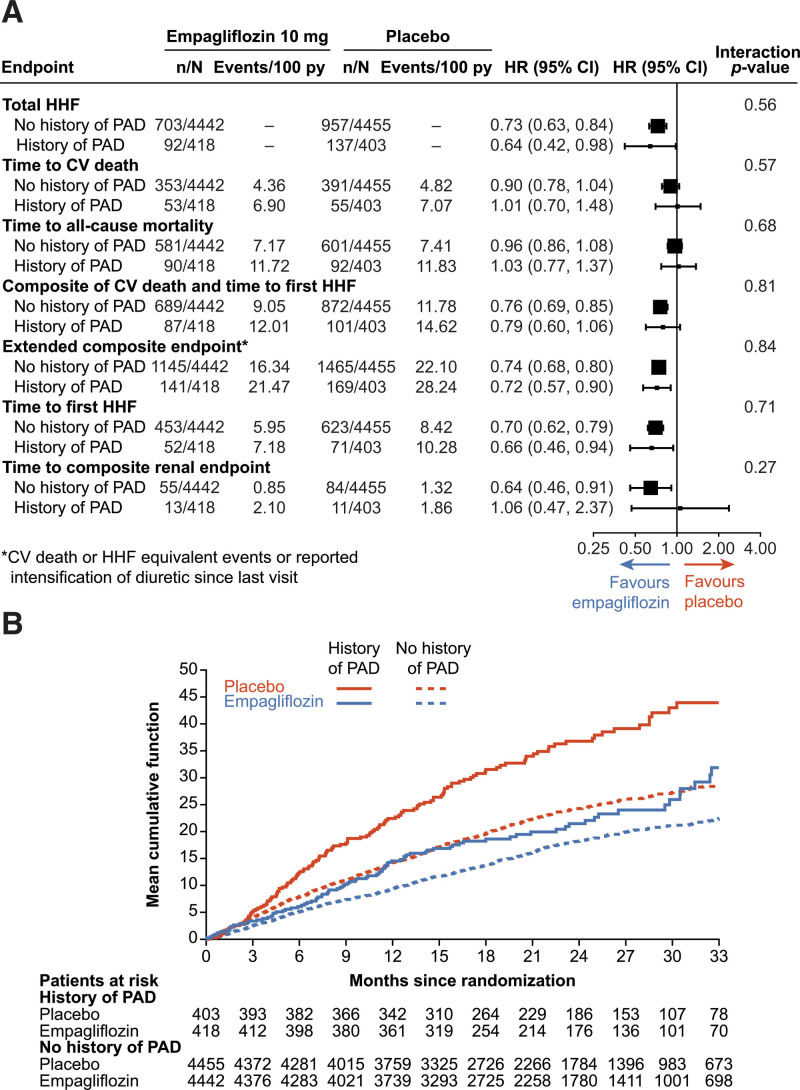
**Summary of major findings.** Panel **A** displays end point data in EMPEROR-Pooled according to peripheral artery disease (PAD) versus no PAD history, and panel **B** displays mean cumulative function of total hospitalizations for heart failure (HHF) in both the placebo and empagliflozin groups by PAD versus no PAD history in EMPEROR-Pooled. EMPEROR-Pooled—a prospectively designed collective analysis of the EMPEROR-Reduced trial (Empagliflozin Outcome Trial in Patients With Chronic Heart Failure and a Reduced Ejection Fraction)^3^ and EMPEROR-Preserved trial (Empagliflozin Outcome Trial in Patients With Chronic Heart Failure With Preserved Ejection Fraction)^4^—included 9718 patients with heart failure across the spectrum of left ventricular ejection fraction (LVEF). Patients were randomized to empagliflozin 10 mg or matching placebo. Outcomes assessed included total HHF, time to first HHF or cardiovascular (CV) death, time to first HHF, time to CV death, and all-cause mortality (ACM). An extended composite outcome of time to first CV death, HHF equivalent event, or intensification of diuretic therapy along with renal end points including the slope of eGFR (estimated glomerular filtration rate) change and a composite renal end point consisting of time to first event of sustained eGFR reduction of ≥50% or end-stage kidney disease (chronic dialysis/renal transplant or sustained eGFR <15 mL/min per 1.73 m^2^ for patients with baseline eGFR ≥30 mL/min per 1.73 m^2^ or sustained eGFR <10 mL/min per 1.73 m^2^ for patients with baseline eGFR <30 mL/min per 1.73 m^2^) or renal death were also presently analyzed. The Kansas City Cardiomyopathy Questionnaire clinical summary scores (KCCQ-CSS), along with adverse events (AEs), were also assessed. The eGFR values presented are derived from the conventional CKD-EPI (Chronic Kidney Disease Epidemiology Collaboration) equation, which utilizes serum creatinine and corrects for age, sex, and race. Institutional review board approval was obtained by each trial center, and informed consent was received from every participant. Baseline characteristics were compared between PAD and no-PAD patients using *t* tests for continuous variables and χ^2^ tests for categorical variables. Time-to-first event analyses were performed with a Cox proportional-hazards model, adjusted for age, sex, region (North America, Latin America, Europe, Asia, and Other), diabetes status (diabetes, prediabetes, no diabetes), eGFR at baseline, LVEF at baseline, and study. These analyses were performed according to the intention-to-treat principle for all randomized patients and included data up to the end of the planned treatment period. Event rates per 100 patient-years and adjusted hazard ratios are reported. Total (first and recurrent) HHF was evaluated accordingly with a joint frailty model that accounted for CV death, adjusted for the same covariates as the Cox model. Continuous end points were analyzed with the same covariates as the Cox model, in addition to visit by treatment by PAD status interaction and baseline value by visit interaction, in a mixed model with repeated measures. To assess the consistency of the treatment effects across PAD and no-PAD subgroups, subgroup-by-treatment interaction terms were added to the models. AEs were analyzed descriptively based on patients with events occurring during the on-treatment period (including 7 days after the last drug consumption by the patient); however, for lower limb amputations, all events up to study completion are shown.

Placebo rates of safety outcomes were higher in people with PAD versus those without PAD in EMPEROR-Pooled, but there was no increase in those treated with empagliflozin compared with placebo (adverse events; PAD: empagliflozin, 89.2%; placebo, 89.1%; no PAD: empagliflozin, 81.5%; placebo, 82.9% and serious adverse events; PAD: empagliflozin, 56.0%; placebo, 62.9%; no PAD: empagliflozin, 44.4%; placebo, 49.1%). Rates of lower limb amputations were higher in people with PAD; however, rates were comparable in empagliflozin- versus placebo-treated patients (PAD: empagliflozin, 2.9%; placebo, 3.5%; no PAD: empagliflozin, 0.4%; placebo, 0.4%).

In this large, contemporary cohort of patients with HF (with either reduced or preserved ejection fraction), we report a significantly elevated risk of HF outcomes among patients with PAD compared with those without PAD, including a ≈50% increase in total HHF and ≈40% increase in cardiovascular and all-cause mortality. While empagliflozin was efficacious in both populations, people with PAD had a higher absolute risk reduction on total HHF events compared with those without PAD. There was no excess in adverse events with empagliflozin in people with PAD, and specifically rates of lower limb amputations, which have been a previous concern with canagliflozin,^5^ were similar. These data underscore an important and previously underappreciated association of PAD with HF events.

## Data Sharing

To ensure independent interpretation of clinical study results and enable authors to fulfill their role and obligations under the ICMJE (International Committee of Medical Journal Editors) criteria, Boehringer Ingelheim grants all external authors access to relevant clinical study data. In adherence with the Boehringer Ingelheim Policy on Transparency and Publication of Clinical Study Data, scientific and medical researchers can request access to clinical study data after publication of the primary manuscript and secondary analyses in peer-reviewed journals and regulatory and reimbursement activities are completed, normally within 1 year after the marketing application has been granted by major regulatory authorities. Researchers should use the https://vivli.org/ link to request access to study data and visit https://www.mystudywindow.com/msw/datasharing for further information.

## Article Information

### Affiliations

### Acknowledgments

The authors meet criteria for authorship as recommended by the International Committee of Medical Journal Editors.

### Sources of Funding

This study was supported by the Boehringer Ingelheim and Eli Lilly and Company Diabetes Alliance.

### Disclosures

S. Verma holds a Tier 1 Canada Research Chair in Cardiovascular Surgery and reports receiving research grants and speaking honoraria from Amarin, Amgen, AstraZeneca (AZ), Bayer, Boehringer Ingelheim (BI), Bristol-Myers Squibb (BMS), Eli Lilly, EOCI Pharmacomm, Ltd, HLS Therapeutics, Janssen, Merck, Novartis, Novo Nordisk (NN), Sanofi, Sun Pharmaceuticals, PhaseBio, and the Toronto Knowledge Translation Working Group. He is a member of the Scientific Excellence Committee of the EMPEROR-Reduced trial and served as a national lead investigator of the DAPA-HF and EMPEROR-Reduced trials. The salary of M.P. Bonaca is partially supported through funds from CPC—a nonprofit academic research organization affiliated with the University of Colorado that receives research grant/consulting funding from Abbott, Agios, Alexion Pharma, Alnylam, Amgen, Angionetics, ARCA Biopharma, Array, AZ, Atentiv, Audentes, Bayer, Better Therapeutics, Brigham and Women’s Hospital, BMS, Cardiol Therapeutics, CellResearch, Cook Medical, Cook, CSL Behring, Eidos Therapeutics, EP Trading Co, Esperion Therapeutics, EverlyWell, Faraday, Fortress Biotech, HDL Therapeutics, Heartflow, Hummingbird Bioscience, Insmed, Janssen, Kowa Research, Lexicon, Merck, Medtronic, Moderna, Novate Medical, NN, Pfizer, PhaseBio, PPD Development, Prairie Education and Research, Prothena Biosciences, Regeneron, Regio Biosciences, Sanifit Therapeutics, Sanofi, Smith and Nephew, Stealth BioTherapeutics, University of Colorado, Worldwide Clinical Trials, Wraser, and Yale Cardiovascular Research Group. He also reports stock in Medtronic and Pfizer and consulting fees from Audentes. J. Butler reports research support from the National Institutes of Health, Patient Centered Outcomes Research, and the European Union. He serves on the speakers’ bureau for Novartis, Janssen, and NN. He serves as a consultant and serves on the Steering Committee, Clinical Events Committee, or data safety monitoring boards for Abbott, Adrenomed, Amgen, Array, AZ, Bayer, Berlin-Cures, BI, BMS, Cardiocell, CVRx, G3 Pharmaceutical, Innolife, Janssen, Lantheus, LinaNova, Luitpold, Medscape, Medtronic, Merck, Novartis, NN, Relypsa, Roche, Sanofi, Stealth-Peptide, SC Pharma, V-Wave, Ltd, Vifor, and ZS Pharma. S.D. Anker reports grants and personal fees from Vifor International and Abbott Vascular and personal fees from AZ, Bayer, Brahms, BI, Cardiac Dimensions, Novartis, Occlutech, Servier, and Vifor International. J. Pedro Ferreira is a consultant for BI. G. Filippatos reports lecture fees and committee member contributions in trials sponsored by Bayer, Medtronic, Vifor, Servier, Novartis, Amgen, and BI and research support from the European Union. J.L. Januzzi is a Trustee of the American College of Cardiology; a board member of Imbria Pharmaceuticals; has received grant support from Applied Therapeutics, Innolife, Novartis Pharmaceuticals, and Abbott Diagnostics; has received consulting income from Abbott, Janssen, Novartis, and Roche Diagnostics; and participates in clinical end point committees/data safety monitoring boards for Abbott, AbbVie, Amgen, Bayer, CVRx, Janssen, MyoKardia, and Takeda. C.S.P. Lam reports research support from Bayer, NN, and Roche Diagnostics; fees as consultant or on the Advisory Board/Steering Committee/Executive Committee for Actelion, Alleviant Medical, Allysta Pharma, Amgen, AnaCardio AB, Applied Therapeutics, AZ, Bayer, BI, Boston Scientific, Cytokinetics, Darma, Inc, EchoNous, Inc, Eli Lilly, Impulse Dynamics, Intellia Therapeutics, Ionis Pharmaceutical, Janssen Research and Development LLC, Medscape/WebMD Global LLC, Merck, Novartis, NN, Prosciento, Inc, Radcliffe Group, Ltd, Recardio, Inc, ReCor Medical, Roche Diagnostics, Sanofi, Siemens Healthcare Diagnostics, and Us2.ai; and position as cofounder and nonexecutive director at Us2.ai. N. Sattar reports personal fees from Abbott Laboratories, Afimmune, Amgen, Eli Lilly, Hanmi Pharmaceuticals, Janssen, Merck Sharp & Dohme, NN, Pfizer, and Sanofi and grants and personal fees from AZ, BI, Novartis, and Roche Diagnostics. T. Iwata, M. Nordaby, and M. Brueckmann are employees of BI. S.J. Pocock is a consultant for BI. M. Packer reports personal fees from Abbvie, Actavis, Amarin, Amgen, AZ, BI, Caladrius, Casana, CSL Behring, Cytokinetics, Imara, Lilly, Moderna, Novartis, Reata, Relypsa, and Salamandra. The other authors report no conflicts.
